# Neuronal autophagy controls excitability via ryanodine receptor–mediated regulation of calcium-activated potassium channel function

**DOI:** 10.1073/pnas.2413651122

**Published:** 2025-04-23

**Authors:** Gaga Kochlamazashvili, Aarti Swaminathan, Alexander Stumpf, Amit Kumar, York Posor, Dietmar Schmitz, Volker Haucke, Marijn Kuijpers

**Affiliations:** ^a^Molecular Pharmacology and Cell Biology, Leibniz-Forschungsinstitut für Molekulare Pharmakologie, Berlin 13125, Germany; ^b^NeuroCure Cluster of Excellence, Charité Universitätsmedizin Berlin, Corporate Member of Freie Universität Berlin, Humboldt-Universität zu Berlin, and Berlin Institute of Health, Berlin 10117, Germany; ^c^Faculty of Biology, Chemistry, Pharmacy, Freie Universität Berlin, Berlin 14195, Germany; ^d^Donders Institute for Brain, Cognition and Behaviour and Faculty of Science, Radboud University, Nijmegen 6525AJ, The Netherlands

**Keywords:** autophagy, neuronal excitability, action potential, potassium channel, ryanodine receptor

## Abstract

Glutamate-mediated neuronal hyperexcitation plays a causative role in eliciting seizures and promoting epileptogenesis. Recent data suggest that altered autophagy can contribute to the occurrence of epilepsy. We examined the role of autophagy in neuronal physiology by generating knockout mice conditionally lacking the essential autophagy protein ATG5 in glutamatergic neurons. We demonstrate that conditional genetic blockade of neuronal autophagy results in action potential narrowing, axonal hyperexcitability, and an increase in kainate-induced epileptiform bursts ex vivo, indicative of a lower threshold for the induction of epileptic seizures. Neuronal hyperexcitability in hippocampal slices from conditional ATG5 knockout mice is due to elevated activity of the large conductance calcium-activated potassium channel BKCa downstream of calcium influx via the endoplasmic reticulum (ER)-localized calcium channel ryanodine receptor (RYR). Consistently, pharmacological blockade of RYR or BKCa function rescued hyperexcitability and reduced the frequency of kainate-induced epileptiform bursts in ATG5 cKO brain slices. Our findings reveal a physiological role for neuronal autophagy in the regulation of neuronal excitability via the control of RYR-mediated calcium release, and thereby, calcium-activated potassium channel function in the mammalian brain.

## Significance

Autophagy is a cellular self-degradation process that is of importance for the health of nerve cells and the brain, in particular during aging. How precisely autophagy contributes to brain health remains debated. We show that impairment of autophagy causes nerve cells of the central nervous system to be hyperexcitable resulting in aberrant network activity that is often seen in animal models of epilepsy and in patients. We further demonstrate that the increased activity of a large conductance potassium channel referred to as BKCa is responsible for the hyperexcitability of nerve cells. Pharmacological blockade of this channel can restore normal neuronal network function and may thus prove to be of relevance for combatting human brain diseases related to impaired autophagy.

Macroautophagy (hereafter referred to simply as autophagy) entails the formation of double-membrane vesicles that deliver dysfunctional proteins and organelles to lysosomes for degradation. The formation of these vesicles termed autophagosomes, is driven by a group of evolutionarily conserved autophagy-related genes (ATG) and proteins. Given that the lifespan of postmitotic neurons can last many decades, degradation and self-renewal processes triggered by autophagy are critical for brain function and neuronal survival. Defects in autophagy have been extensively reported in neurodegenerative disorders that are characterized by impaired degradation and the abnormal accumulation of protein aggregates including for example Alzheimer’s disease, Huntington’s disease, and amyotrophic lateral sclerosis (1). Recent data show that autophagy also plays important roles in regulating synaptic function and neuronal activity (2–5). At the postsynaptic site, autophagy has been reported to modulate neurotransmission via the degradation of ionotropic glutamate receptors, γ-aminobutyric acid (GABA) receptors, and synaptic scaffolding proteins (6–9). Recent work from us (2) and others (10, 11) has revealed a key role for neuronal autophagy in the control of the axonal tubular endoplasmic reticulum (ER) (i.e., a process referred to as ER-phagy), and, thereby, in presynaptic neurotransmission. Loss of autophagy resulted in facilitated excitatory neurotransmission via altered presynaptic calcium buffering due to an increase in the levels of axonal ryanodine receptors (RYRs) that mediate the release of calcium ions from the ER. Conversely, upregulation of neuronal ER-phagy has been shown to improve organismal fitness (12). Finally, alterations in the autophagy pathways caused by changes in the activity of the mechanistic target of rapamycin (mTOR) pathway (13–15), aggregation of abnormal proteins (16), or genetic alterations in ATGs (14, 17, 18) or other genes involved in autophagy (18–22) have been implicated in epileptogenesis. Neuronal hyperactivity or epileptiform activities may conceivably contribute to neurodegeneration and memory deficits found in autophagy-deficient animal models and in aging humans. How impaired autophagy causes epilepsy remains unclear.

Here, we report that increased activity of large conductance calcium-activated potassium (BKCa) channels causes increased neuronal excitability in autophagy-deficient ATG5 conditional knockout (KO) mice. BKCa channels are voltage-gated potassium channels localized to the axon and to nerve terminals (23) that conduct large amounts of potassium ions and can be activated by calcium elevation downstream of RYR function via double-nanodomain coupling between somatic plasma membrane and axonal ER cisternae (24–26). Modulation of BKCa channel function has been implicated in regulating neuronal firing properties, action potential waveform, and fast afterhyperpolarization (fAHP) (27–31). Dysfunction of BKCa channels has been shown to contribute to the pathophysiology of various neurological disorders including epilepsy, movement disorders, and neurodevelopmental and cognitive disorders (29, 32). Genetic studies have shown that BKCa gain-of-function mutations facilitate repolarization or hyperpolarization and, thereby, enable faster recovery of sodium channels to promote high frequency firing (33, 34). BKCa channels have thereby emerged as a drug target for the treatment of epilepsy.

We demonstrate that neuronal hyperexcitability in hippocampal slices from conditional ATG5 knockout mice is due to elevated BKCa channel activity downstream of calcium influx via ER-localized RYRs that accumulate in ATG5-KO axons. Our findings establish a link between autophagy and neuronal hyperexcitability and reveal a critical role for ER calcium release in the modulation of neuronal excitability.

## Results

### Epileptiform Bursts and Increased Excitability of Pyramidal Neurons in Autophagy-Deficient ATG5 cKO Mice.

**Fig. 1.**
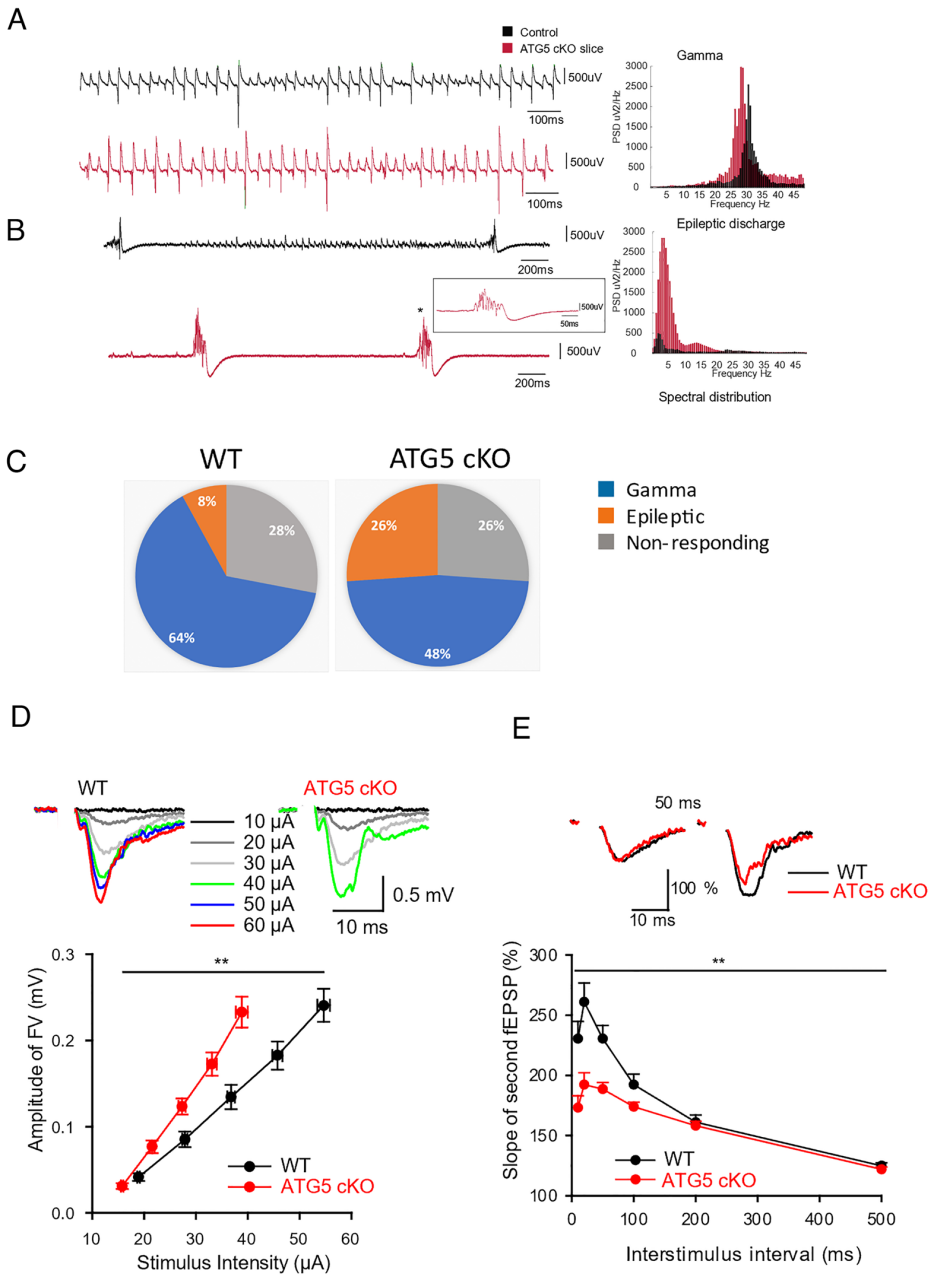
Epileptiform bursts and increased excitability of pyramidal neurons in autophagy-deficient ATG5 cKO mice. (*A*–*C*) Kainate-induced gamma oscillations and epileptic discharges recorded from CA3b region of hippocampal slices in wildtype control and ATG5 cKO mice. (*A*) 1.5 s LFP trace of 300 nM kainate-induced gamma oscillation recorded in wildtype hippocampal slice (in black) and ATG cKO hippocampal slice (in red). The *Right* panel shows the power spectral distributions of 60 s trace. Note the peak of the power spectral density (PSD) for wildtype control at ~31 Hz (in black) and for ATG5 cKO at ~29 Hz (in red) indicating gamma oscillations (*SI Appendix*, Fig. S1*A*). (*B*) Last 3 s trace after wash-in of 300 nM kainate to induce epileptic discharges recorded in wildtype control slice (in black) and ATG cKO hippocampal slice (in red). *Inset*: an epileptic discharge event at higher resolution (marked in asterisk). The *Right* panel shows the power spectral distributions of 60 s trace of epileptic discharges for wildtype control and for ATG5 cKO. (*C*) Fraction of slices displaying gamma (blue), epileptic discharges (orange), or no response (gray) in kainate-induced recordings (wildtype control n = 25; ATG5 cKO n = 23). There were significantly more slices with epileptic discharges in ATG5 cKO mice compared to that of wildtype control mice (*P* = 0.046; z = −1.68, two-sample proportion test; one-tailed). Moreover, more epileptic events were detected per slice in ATG5 cKO compared to controls (control n = 113 events vs ATG KO n = 727 events; see *SI Appendix*, Fig. S1*D*). (*D*) Increased excitability of Schaffer collaterals in ATG5 cKO mice. Increasing stimulation intensities applied to Schaffer collaterals induced facilitated FV amplitudes in ATG5 cKO mice (two-way RM ANOVA, *P* = 0.005; WT n = 14, N = 8; ATG5 cKO n = 13, N = 8). *Inserts*: Representative CA1-fEPSPs recorded in response to increasing stimulation intensities in control and in ATG5 cKO mice. (*E*) Reduced paired-pulse facilitation (PPF) in ATG5 cKO mice. PPF ratios quantified over a range of interstimulus intervals (10 to 500 ms), given as a percentage increase of the second response in relation to the first, show reduced facilitation of the second response in ATG5-cKO mice (two-way RM ANOVA, *P* = 0.004). *Inserts*: Representative traces of fEPSP-PPF at a 50-ms interstimulus in control and ATG5-cKO mice show reduced PPF, indicative of increased initial release probability in ATG5 cKO mice. The number of tested slices n and mice N are as reported in Fig. 1*D*.

Previous work revealed that Emx1-Cre-induced conditional loss of ATG5 in cortical and hippocampal excitatory neurons potently reduced the stimulus strength required to elicit saturated fEPSP responses compared to wild-type (WT) littermate controls (2). We reasoned that this comparably large electrophysiological phenotype in extracellular recordings and the severely impaired postnatal viability of ATG5 cKO mice may not be fully explained by the modest alteration in presynaptic release probability reported (2). We hypothesized that block of neuronal autophagy in absence of ATG5, in addition to its effects on release probability, may affect the intrinsic excitability of hippocampal neurons and, thereby, lead to epileptic discharges that conceivably could explain the poor survival of ATG5 cKO mice. To test this, we recorded gamma oscillations and epileptiform discharges (Fig. 1 *A* and *B*) induced by bath application of the excitatory neurotoxin kainate, in the CA3b area of hippocampal slice preparations (35) from WT and ATG5 cKO mice. The fraction of slices displaying kainate-induced epileptic discharges was dramatically increased in ATG5 cKO mice (26%) compared to WT controls (8%), (Fig. 1*C*). Moreover, quantification of epileptic discharges in slices from both genotypes revealed the interevent intervals (IEI) of epileptic events in ATG5 cKO slices to be significantly different from control slices (*SI Appendix*, Fig. S1*D*; KS test: *P* = 0.0002). The peak frequencies of gamma oscillations (36) at maximum power recorded in CA3b were significantly lower in ATG5 cKO slices compared to that of controls (*SI Appendix*, Fig. S1*A*; Control 31.5 ± 0.57 Hz vs ATG cKO 29.84 ± 0.33 Hz; *P* = 0.011, unpaired *t* test). Other attributes of gamma oscillations such as gamma power at maximum frequency or the area under the curve of the power spectral density were unaffected by genotype (*SI Appendix*, Fig. S1 *B* and *C*). Loss of ATG5 in excitatory neurons thus causes an increase in kainate-induced epileptiform bursts ex vivo, indicative of a lower threshold for the induction of epileptic seizures.

Given the selective accumulation of tubular ER in the axonal and presynaptic compartments of ATG5 cKO mice (2), we reasoned that the observed epileptiform discharges might be due to altered axonal excitability. We therefore recorded input/output stimulus response curves of CA1 fEPSPs as a measure of basal excitatory synaptic transmission and excitability of presynaptic fibers by measuring fiber volley (FV) amplitudes. To exclude altered GABAergic inhibitory modulation of excitatory neurotransmission and neuronal excitability in ATG5 cKO mice, we applied the GABAergic antagonist Picrotoxin. Moreover, we separated the presynaptic axonal compartments from their cell bodies by dissecting CA3-CA1 connections (*SI Appendix*, Fig. S1*E*) and measured FV amplitudes as a proxy for axonal excitability. We found that FV amplitudes were significantly facilitated in ATG5 cKO compared to WT control mice in response to increasing stimulation intensities (Fig. 1*D*) and much lower stimulation intensities were required to elicit maximal FV amplitudes (Fig. 1*D*; ATG5-cKO: 38.84 ± 1.15 µA; WT: 54.64 ± 1.22 µA). Increased FV excitability was also overt from a comparison of fEPSP slopes as a function of FV amplitudes (*SI Appendix*, Fig. S1*F*) *vs.* stimulus intensity (*SI Appendix*, Fig. S1*G*), with the latter revealing a much larger difference between genotypes. These data from extracellular recordings indicate that postsynaptic fEPSP responses in ATG5 cKO mice (*SI Appendix*, Fig. S1*G*) are facilitated by a compound increase of FV (i.e., axonal) excitability (Fig. 1*D*) and basal excitatory neurotransmission (*SI Appendix*, Fig. S1*F*) as further evidenced by reduced paired-pulse facilitation (PPF) of fEPSP slopes (Fig. 1*E*).

Taken together our findings show that conditional blockade of neuronal autophagy in addition to increased presynaptic release probability (2) (Fig. 1*E* and *SI Appendix*, Fig. S1*F*) also leads to increased excitability (Fig. 1*D* and *SI Appendix*, Fig. S1*G*), resulting in epileptiform burst activity (Fig. 1 *A*-*C*).

### Hyperexcitability and Increased Release Probability in ATG5 cKO Mice are Both Caused by Elevated RYR Function.

**Fig. 2.**
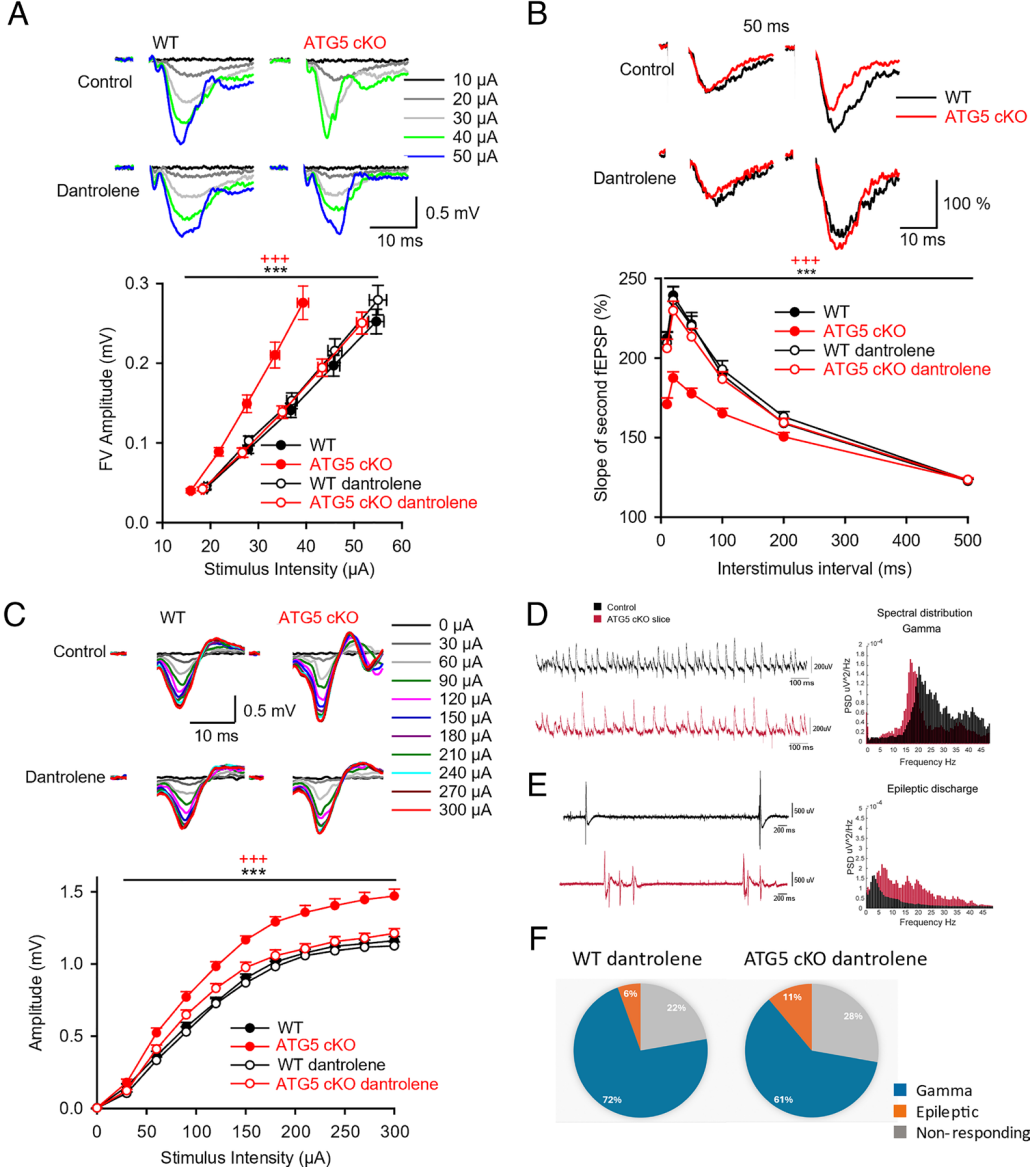
RYR inhibition in presence of Dantrolene rescues neuronal hyperexcitability caused by conditional loss of ATG5. (*A*) Facilitated FV excitability in ATG5 cKO mice is rescued with RYR inhibitor Dantrolene. Increasing stimulation intensities applied to Schaffer collaterals induced facilitated FV amplitudes recorded in CA1 area of ATG5 cKO mice, indicating increased excitability of Schaffer collaterals (two-way RM ANOVA, *P* < 0.001; WT n = 14, N = 5; ATG5 cKO n = 15, N = 5). Facilitated FV amplitudes in ATG5 cKO mice are rescued with RYR inhibitor Dantrolene (two-way RM ANOVA, *P* < 0.001; ATG5 cKO Dantrolene n = 12, N = 5). RYR inhibition has no further effect on WT responses, compared to untreated WT slices (two-way RM ANOVA, *P* = 0.337; WT Dantrolene n = 13, N = 5) and WT vs ATG5 cKO slices treated with Dantrolene show no significant difference between genotypes (two-way RM ANOVA, *P* = 0.678). *Inserts*: Representative CA1-fEPSPs recorded in response to increasing stimulation intensities in control and treated with RYR inhibitor Dantrolene. Facilitated CA1-fEPSPs induced by stimulation of Schaffer collaterals in ATG5 cKO mice are rescued when treated with Dantrolene (2 h–30 µM). (*B*) Reduced PPF in ATG5 cKO mice is rescued with RYR inhibitor Dantrolene. PPF ratios quantified over a range of interstimulus intervals (10 to 500 ms), given as a percentage increase of the second response in relation to the first, show reduced facilitation of the second response in ATG5-cKO mice (two-way RM ANOVA, *P* < 0.001). Reduced PPF in ATG5 cKO mice are rescued with RYR inhibitor Dantrolene (two-way RM ANOVA, *P* < 0.001). RYR inhibition has no effect on WT responses, compared to untreated WT slices (two-way RM ANOVA, *P* = 0.977) and WT vs ATG5 cKO slices treated with Dantrolene show no significant difference between genotypes (two-way RM ANOVA, *P* = 0.506). *Inserts*: Representative traces of fEPSP-PPF at a 50-ms interstimulus in control and treated with RYR inhibitor Dantrolene. Reduced PPF in ATG5 cKO mice is rescued with Dantrolene (2 h–30 µM). The number of tested slices n and mice N are as reported in Fig. 2*A*. (*C*) Facilitated CA3 excitability in ATG5 cKO mice is rescued with RYR inhibitor Dantrolene. Increasing stimulation intensities (from 0 to 300 μA with a 30 μA step) applied to Schaffer collaterals induced bigger population spikes (PS) in CA3 area in ATG5 cKO mice indicating increased cellular excitability of CA3 excitatory neurons (two-way RM ANOVA, *P* < 0.001; WT n = 15, N = 5; ATG5 cKO n = 15, N = 5). Facilitated CA3 excitability in ATG5 cKO mice is rescued with RYR inhibitor Dantrolene (two-way RM ANOVA, *P* < 0.001; ATG5 cKO Dantrolene n = 15, N = 5). RYR inhibition has no effect on WT responses, compared to untreated WT slices (two-way RM ANOVA, *P* = 0.306; WT Dantrolene n = 15, N = 5) and WT vs ATG5 cKO slices treated with Dantrolene show no significant difference between genotypes (two-way RM ANOVA, *P* = 0.052). *Inserts*: Representative CA3-PS traces recorded in response to increasing stimulation intensities in control and with RYR inhibitor Dantrolene. Facilitated CA3-PS amplitudes, induced by antidromic stimulation in ATG5 cKO mice are rescued with Dantrolene (2 h–30 µM). (D-F) Kainate-induced gamma oscillations and epileptic discharges recorded from CA3b region of hippocampal slices incubated with Dantrolene (30 µM) in wildtype control and ATG5 cKO mice. (*D*) 1.5 s LFP trace of 300 nM kainate-induced gamma oscillation recorded in wildtype hippocampal slice (in black) and ATG cKO hippocampal slice (in red). The *Right* panel shows the power spectral distributions of 60 s trace. (*E*) Last 5 s trace after wash-in of 300 nM kainate to induce epileptic discharges recorded in wildtype control slice (in black) and ATG cKO hippocampal slice (in red) incubated with Dantrolene (30 µM). The *Right* panel shows the power spectral distributions of 60 s trace of epileptic discharges for wildtype control and for ATG5 cKO. (*F*) Fraction of slices displaying gamma (blue), epileptic discharges (orange), or no response (gray) in kainate-induced recordings (wildtype control n = 18; ATG5 cKO n = 18). The number of slices with epileptic discharges in ATG5 cKO mice was not significantly different compared to that of wildtype control mice after 30 µM Dantrolene treatment (*P* = 0.2742; z = −0.603, two-sample proportion test; one-tailed).

We hypothesized that the increased excitability of pyramidal neurons may be due to the upregulation of axonal RYR levels and function in ATG5 cKO hippocampal neurons (2). We tested this by preincubating slices with the RYR antagonist Dantrolene. FV amplitudes, an indicator of axonal excitability, were significantly facilitated in ATG5 cKO mice in response to increasing stimulation intensities in control slices treated with DMSO (Fig. 2*A*). Pharmacological inactivation of RYRs by Dantrolene efficiently rescued FV amplitudes in ATG5 cKO mice to those measured in WT controls (Fig. 2*A*). RYR inhibition did not affect FV amplitudes of WT responses. Elevated postsynaptic fEPSP slopes as a function of FV amplitudes (*SI Appendix*, Fig. S2*A*) or stimulus intensity (*SI Appendix*, Fig. S2*B*) were both rescued by Dantrolene-mediated RYR inhibition, whereas Dantrolene was without effect in slices from WT animals. We then analyzed paired pulse facilitation (PPF) of fEPSPs taken as a surrogate measure for presynaptic release probability (37), in the absence or presence of Dantrolene. Dantrolene application fully rescued the reduced initial PPF values in ATG5 cKO slices to the level observed in WT controls (Fig. 2*B*). These data suggest that increased RYR levels and activity causally underlie the elevated released probability [consistent with our earlier data; (2)] as well as the hyperexcitability (Figs. 1 and 2*A*) of pyramidal neurons from ATG5 cKO mice. The fact that Dantrolene-mediated RYR inhibition fully rescued presynaptic hyperexcitability and elevated release probability of ATG5 cKO neurons, predicts that the facilitated compound fEPSP slopes should also be restored to WT levels. Indeed, analysis of fEPSP slopes in response to increasing stimulation intensities revealed a complete rescue of postsynaptic fEPSP slopes in ATG5 cKO slices by Dantrolene (*SI Appendix*, Fig. S2*B*).

We challenged these results of fEPSP excitability by measuring cellular excitability of CA3 excitatory neurons using direct antidromic stimulation of CA3 axons and measuring CA3-population spike (PS) amplitudes (*SI Appendix*, Fig. S2*C*). We applied increasing stimulation intensities from 0 to 300 µA and found that ATG5 cKO in contrast to WT slices, displayed significantly higher CA3-PS amplitudes in control conditions (Fig. 2*C*). Incubation of ATG5 cKO slices with Dantrolene effectively rescued elevated CA3-PS amplitudes in ATG5 cKO slices to WT levels (Fig. 2*C*). CA3-PS amplitudes in WT slices were unaffected by Dantrolene.

Finally, we examined the effects of Dantrolene on kainate-induced gamma oscillations and seizure-like discharges. As explained above, ATG5 cKO slices exhibited a higher fraction of epileptic discharges and a reduced gamma frequency compared to WT (Fig. 1*C* and *SI Appendix*, Fig. S1*A*). Following Dantrolene treatment, the proportion of slices exhibiting epileptic events in ATG5 KO decreased from 26 to 11%, reaching levels comparable to WT (6%, Fig. 2 *D*–*F*). Moreover, gamma oscillation peak frequency was significantly reduced in both WT and ATG5 KO slices after Dantrolene exposure, bringing them to similar levels (*SI Appendix*, Fig. S2*D*). Importantly, the differences in epileptic event occurrence between WT and ATG5 cKO slices seen in nontreated controls were no longer significant in the presence of Dantrolene, indicating that Dantrolene restored network excitability in ATG5 cKO slices to WT levels.

Collectively, these data indicate that hyperexcitability and elevated release probability in ATG5 cKO pyramidal neurons are caused by increased RYR levels and function.

### Increased Excitability and Shorter AP Waveform in ATG5 cKO Pyramidal Neurons.

**Fig. 3.**
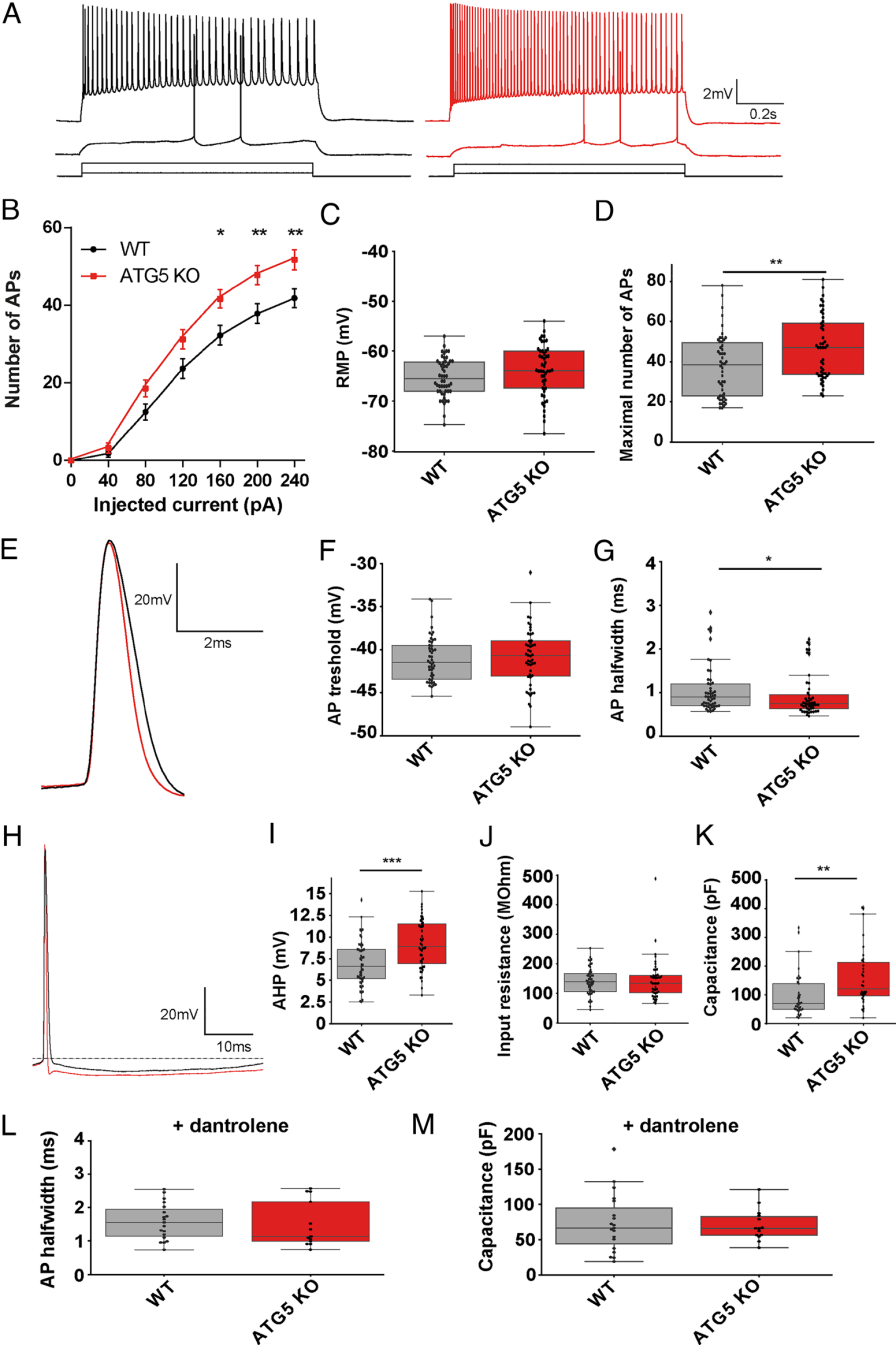
Increased excitability and shorter AP waveform in ATG5 cKO pyramidal neurons. (*A*) Example traces of cell responses during depolarization steps (*Top*: 200pA, *Bottom*: 80 pA) in CA1 pyramidal neurons in WT (black) and ATG5 cKO (red) animals. Scale bar corresponds to 20 mV/0.2 s. (*B*) Number of action potential (AP)/current intensity relationship displays significant increase in firing frequency in ATG5 cKO animals for higher current intensities. WT: n = 28; ATG5 cKO n = 26; two-way-ANOVA *P* = 0.0094; Sidak’s test for multiple comparison. Error bars represent mean ± SEM. (*C*) Resting Membrane Potential (RMP) was not significantly different in ATG5 deficient cells; Student’s *t* test *P* = 0.107. (*D*) Maximal firing rate during 1 s-depolarization pulse, before cells went into depolarization-block. Student’s *t* test: *P* = 0.006. (*E*) Example traces of a single AP waveform of WT (black) and ATG5 deficient (red) CA1 pyramidal cells. Scale bar corresponds to 20 mV/2 ms. (*F*) AP threshold is not changed in ATG5 cKO compared to WT cells; Student’s *t* test p = 0.47. (*G*) AP halfwidth is reduced in ATG5 deficient cells (mean ± SEM AP halfwidth for WT = 1.076 ± 0.078 ms, n = 50; mean ± SEM AP halfwidth for ATG5 KO = 0.937 ± 0.072 ms, n = 48); Mann–Whitney test: *P* = 0.027. (*H*) APs from WT (black) and ATG5 deficient (red) cells are superimposed to compare their peak AHP. (*I*) ATG5 depletion significantly increased peak AHP (mean ± SEM AHP for WT = 6.944 ± 0.37 mV, n = 50; mean ± SEM AHP for ATG5 KO = 9.198 ± 0.41 mV, n = 48; *P* = 0.00016) but (*J*) did not change input resistance; Mann–Whitney test *P* = 0.0.704. (*K*) ATG5 depletion significantly increased capacitance (mean ± SEM capacitance for WT = 98.02 ± 12.52 pF, n = 37; mean ± SEM capacitance for ATG5 KO = 158.48 ± 17.26 pF; *P* = 0.001). (*L*) Application of the RYR inhibitor Dantrolene (30 µM) rescued the reduced AP halfwidth (mean ± SEM AP halfwidth for WT in presence of dantrolene = 1.577 ± 0.124 ms, n = 19; mean ± SEM AP halfwidth for ATG5 KO in presence of dantrolene = 1.494 ± 0.189 ms, n = 13; *P* = 0.57) and (*M*) change in capacitance in ATG5 cKO mice (mean ± SEM capacitance for WT in presence of dantrolene = 73.253 ± 9.54 pF, n = 19; mean ± SEM capacitance for ATG5 KO in presence of dantrolene = 70.575 ± 6.17 pF, n = 13; *P* = 0.96).

The amplitudes of presynaptic fiber volleys (FV) were plotted against stimulus intensity for measures of presynaptic Schaffer collateral excitability. As presynaptic Schaffer collaterals were dissected from CA3 cell bodies we assume FV amplitudes as a measure of axonal excitability, which is no longer influenced by dendritic and cellular excitability. Next, we aimed to study cellular excitability of excitatory neurons in Atg5 cKO mice using the whole-cell patch-clamp approach. CA1 pyramidal neurons in acute slice preparations from ATG5 cKO mice displayed increased excitability as evidenced by a significantly higher number of APs at current injections of 160 to 240 pA compared to WT neurons (Fig. 3 *A* and *B*). The maximal number of APs during a 1 s depolarization pulse was also elevated in slices from autophagy-deficient ATG5 cKO animals (Fig. 3*D*). In contrast, resting membrane potential (RMP) (Fig. 3*C*), AP threshold (Fig. 3*F*), and input resistance (Fig. 3*J*) did not differ between WT and ATG5 cKO neurons. Cell capacitance was slightly increased in ATG5 cKO neurons (Fig. 3*K*). Further analysis of the AP waveform revealed a shorter AP halfwidth (Fig. 3 *E* and *G*) and increased afterhyperpolarization in ATG5 cKO cells (Fig. 3 *H* and *I*). Detailed analyses of the AP waveform showed that the slope ratios (maximal positive slope/maximal negative slope) were reduced in ATG5 cKO neurons (*SI Appendix*, Fig. S3*A*) as a consequence of increased negative AP slopes (*SI Appendix*, Fig. S3*B*). Positive AP slopes (i.e., depolarizing sodium currents) (*SI Appendix*, Fig. S3*C*) or current-voltage characteristics (*SI Appendix*, Fig. S3*D*) did not differ between genotypes. We probed whether these differences might be related to elevated RYR function in ATG5 cKO neurons. Pharmacological inactivation of RYRs by Dantrolene increased AP halfwidth to values that did not differ significantly between slices from WT and ATG5 cKO mice Fig. 3*L*). Moreover, application of Dantrolene also fully rescued the small increase in capacitance in ATG5 cKO neurons to the level measured in WT controls (Fig. 3*M*).

These results suggest that blockade of autophagy upon conditional loss of ATG5 in excitatory neurons results in increased excitability and shorter AP waveform due to increased potassium-efflux during the AP, suggestive of facilitated repolarizing potassium channel function. The rescue of AP halfwidth by Dantrolene indicates a further role for RYR in shaping excitability.

### Increased Excitability and Kainate-Induced Epileptic Discharges in ATG5 cKO are Largely due to BKCa Channel Hyperactivity.

Given the effect of conditional ATG5 loss on AP waveform and afterhyperpolarization (AHP), processes known to be modulated by the large conductance calcium-activated potassium channel BKCa encoded by KCNMA1 (27, 30, 31, 34, 38), and the functional association of BKCa channels with the RYR (24–26), we focused our further analysis on BKCa. Since ATG5 cKO neurons exhibit increased RyR accumulation in axons (2) (*SI Appendix*, Fig. S3*E*), we hypothesized that enhanced RyR-mediated calcium release leads to increased BKCa activity, contributing to the observed hyperexcitability phenotype.

**Fig. 4.**
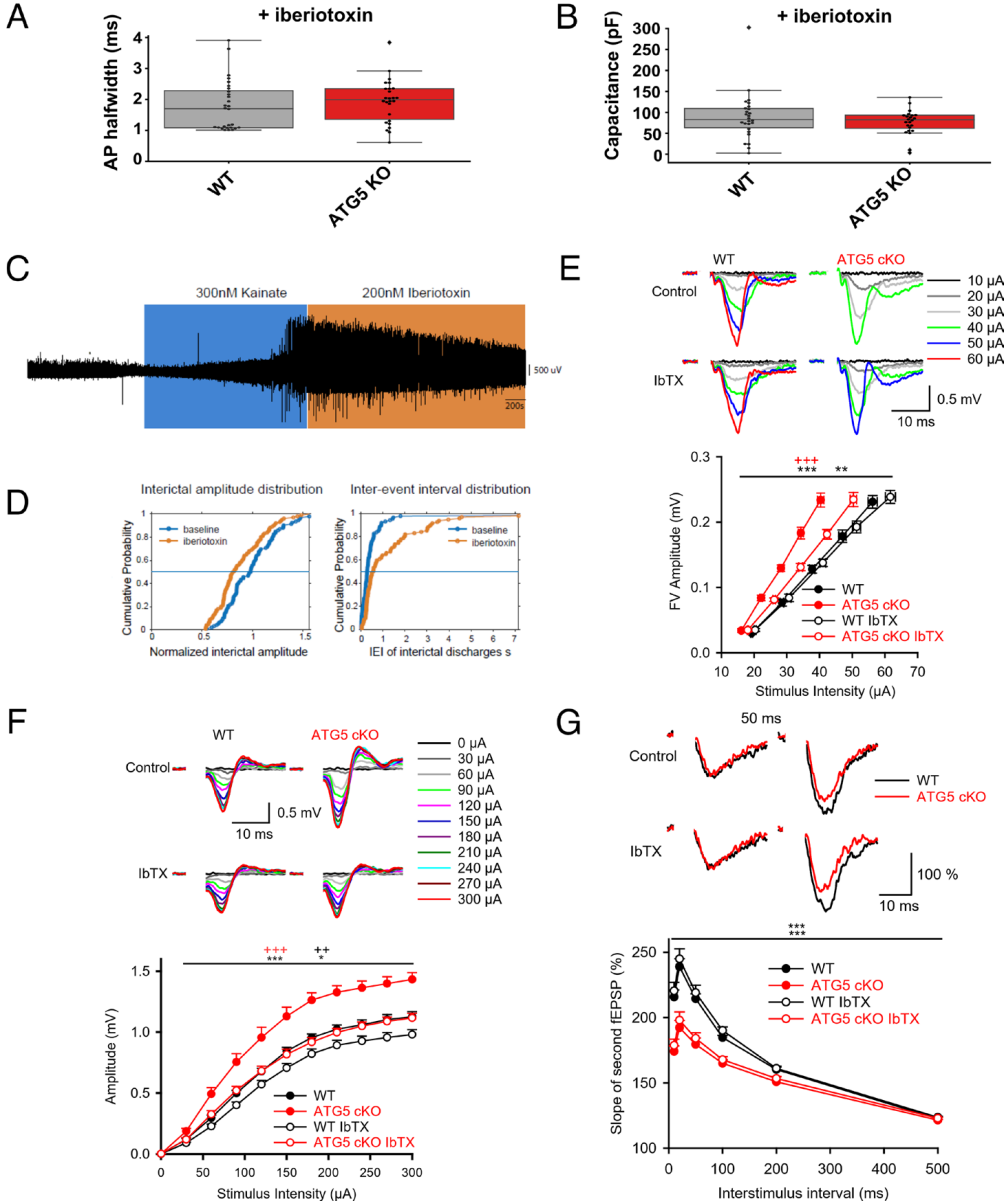
BKCa channel inhibition in presence of Iberiotoxin rescues neuronal hyperexcitability caused by conditional loss of ATG5. (*A*) AP halfwidth (mean ± SEM AP halfwidth for WT in presence of Iberiotoxin = 1.784 ± 0.16 ms, n = 26; mean ± SEM AP halfwidth for ATG5 KO in presence of Iberiotoxin = 1.945 ± 0.138 ms, n = 24) and (*B*) capacitance changes in ATG5 cKO mice (mean ± SEM capacitance for WT in presence of Iberiotoxin = 89.205 ± 11.2 pF, n = 26; mean ± SEM capacitance for ATG5 KO in presence of Iberiotoxin = 91.327 ± 15.81 pF, n = 24) are rescued by the BKCa channel blocker Iberiotoxin (200 nM). Mann–Whitney test: *P* = 0.35 and *P* = 0.60, respectively. (*C*) Effect of BKCa channel blocker Iberiotoxin (100 or 200 nM) on kainate-induced epileptic discharges in ATG5 cKO mouse hippocampal slices. *Insert*: Example of an 84 min LFP recording with baseline (in gray), 300 nM kainate wash-in (in blue) followed by wash-in of 200 nM Iberiotoxin (in orange). Note the development of high-amplitude epileptic discharges after the kainate wash-in and the reduction in amplitude and occurrence of epileptic discharges after wash-in of Iberiotoxin. (*D*) *Left:* Distribution of epileptic discharge amplitudes during last 5 min of baseline (300 nM kainate) and last 5 min of 200 nM Iberiotoxin (n = 6 slices, 92 events; Kolmogorov–Smirnov test: *P* = 0.0021). *Right:* Distribution of epileptic discharge interevent intervals (IEI) during last 5 min of baseline (300 nM kainate) and last 5 min of 200 nM Iberiotoxin (n = 6 slices, 100 events; Kolmogorov–Smirnov test: *P* = 0.0004). (*E*) Facilitated FV excitability in ATG5 cKO mice is partially rescued with BKCa antagonist Iberiotoxin. Increasing stimulation intensities applied to Schaffer collaterals induced facilitated FV amplitudes in ATG5 cKO mice, indicating increased excitability of Schaffer collaterals (two-way RM ANOVA, *P* < 0.001; WT n = 12, N = 6; ATG5 cKO n = 14, N = 7). Facilitated FV amplitudes in ATG5 cKO mice are partially rescued after application of BKCa antagonist Iberiotoxin (two-way RM ANOVA, *P* < 0.001; ATG5 cKO n = 14, N = 7). Iberiotoxin has no effect on WT responses, compared to untreated WT slices (two-way RM ANOVA, *P* = 0.478; WT Iberiotoxin n = 12, N = 6) and WT vs ATG5 cKO slices treated with Iberiotoxin still show a significant difference between genotypes (two-way RM ANOVA, *P* = 0.003). *Inserts*: Representative CA1-fEPSPs recorded in response to increasing stimulation intensities in control and treated with BKCa antagonist Iberiotoxin. Facilitated CA1-fEPSPs induced by stimulation of *Schaffer collaterals* in ATG5 cKO mice are partially rescued when treated with Iberiotoxin (30 to 40 min, 200 nM). (*F*) Facilitated CA3-PS amplitudes induced by antidromic stimulation in ATG5 cKO mice are rescued with Iberiotoxin (30 to 40 min, 200 nM). Increasing stimulation intensities (from 0 to 300 μA with a 30 μA step) applied to Schaffer collaterals induced bigger PSs in CA3 area of ATG5 cKO mice, indicating increased cellular excitability of CA3 cells (two-way RM ANOVA, *P* < 0.001; WT n = 14, N = 7; ATG5 cKO n = 14, N = 7). Facilitated CA3 excitability in ATG5 cKO mice is rescued with BKCa antagonist Iberiotoxin (two-way RM ANOVA, *P* < 0.001; ATG5 cKO Iberiotoxin n = 15, N = 6). Iberiotoxin slightly affected WT responses, somewhat reducing amplitudes of CA3-PSs, compared to untreated WT slices (two-way RM ANOVA, *P* = 0.009; WT Iberiotoxin n = 16, N = 6). WT vs ATG5 cKO slices treated with Iberiotoxin show small but still significant difference between genotypes (two-way RM ANOVA, *P* = 0.019). *Inserts*: Representative CA3-PSs traces recorded in response to increasing stimulation intensities in control and with BKCa antagonist Iberiotoxin. (*G*) Reduced PPF in ATG5 cKO mice is not rescued with BKCa antagonist Iberiotoxin. PPF quantified over a range of interstimulus intervals (10 to 500 ms), given as a percentage increase of the second response in relation to the first, show reduced facilitation of the second response in ATG5 cKO mice (two-way RM ANOVA FV, *P* < 0.001). Reduced PPF in ATG5 cKO mice are not rescued after application of BKCa antagonist Iberiotoxin (two-way RM ANOVA, *P* = 0.410). Iberiotoxin has no effect on WT responses, compared to untreated WT slices (two-way RM ANOVA, *P* = 0.417) and WT vs ATG5 cKO slices treated with Iberiotoxin show a significant difference between genotypes (two-way RM ANOVA, *P* < 0.001). *Inserts*: Representative traces of fEPSP-PPF at a 50-ms interstimulus in control and treated with BKCa antagonist Iberiotoxin. Reduced PPF an indicative of increased initial release probability in ATG5 cKO mice is not rescued with Iberiotoxin. The number of tested slices n and mice N are as reported in Fig. 4*E*.

We capitalized on the fact that BKCa channel function can be potently inhibited by Iberiotoxin (39) to probe whether pharmacological blockade of BKCa channels rescues AP halfwidth, hyperexcitability, and/or increased basal synaptic transmission in ATG5 cKO pyramidal neurons. First, we applied Iberiotoxin in our whole cell patch-clamp recordings to assess its effect on AP halfwidth and capacitance. Iberiotoxin increased the AP halfwidth and reduced afterhyperpolarization in both genotypic groups to similar levels, e.g. rescuing the phenotype of ATG5 cKO (Fig. 4*A* and *SI Appendix*, Fig. S4*A*). In addition, BKCa channel inhibition restored the increased capacitance in ATG5 cKO neurons to WT levels (Fig. 4*B*), similar to the effect observed with Dantrolene treatment (Fig. 3 *L* and *M*). These results suggest that elevated BKCa channel function directly or indirectly underlie the altered AP halfwidth as well as the observed capacitance changes in ATG5 cKO pyramidal neurons.

To determine whether elevated BKCa channel function is also causative for the observed epileptiform bursts in ATG5 cKO neurons, we applied Iberiotoxin after the induction of epileptic discharges by kainate (300 nM). Application of Iberiotoxin significantly reduced kainate-induced epileptic discharges by reducing interictal amplitudes and increasing IEI in ATG5 cKO mice (Fig. 4 *C* and *D*). We then analyzed whether Iberiotoxin-mediated BKCa blockade restores elevated FV excitability by recording fEPSP responses before and 30 to 40 min post application of the drug. Application of Iberiotoxin significantly reduced facilitated FV amplitudes in ATG5 cKO slices (Fig. 4*E*) but had no effect on FV amplitudes of WT responses consistent with earlier reports (27). The remaining small difference between FV amplitudes of slices from both genotypes indicates the existence of Iberiotoxin-insensitive components of increased FV excitability in ATG5 cKO mice, for example via RYR-mediated calcium modulation of other channels by cell signaling. To corroborate these data, we studied the effects of Iberiotoxin on excitability of CA3 neurons by measuring CA3-PS amplitudes in response to antidromic stimulation (*SI Appendix*, Fig. S2*C*). Incubation of ATG5 cKO slices with Iberiotoxin effectively restored facilitated CA3-PS amplitudes in ATG5 cKO slices to those measured in untreated WT slices (Fig. 4*F*). We noted a mild effect of Iberiotoxin on WT responses that may indicate a differential sensitivity of BKCa channels between the axonal and somatic compartments (27), e.g. via different BKCa subunit composition (28).

To test whether the observed gain in BKCa channel function is due to altered BKCa levels, we conducted quantitative proteomic analysis of steady-state protein levels in cerebellar granule cell neurons by stable isotope labeling with amino acids in cell culture (SILAC) experiments. These experiments failed to reveal any significant differences in the expression levels of any of the detectable potassium channels, including BKCa (KCNMA) (*SI Appendix*, Fig. S3*E*). In addition, BKCa labeled with antibodies displayed a punctate distribution in hippocampal neurons from WT or ATG5 cKO mice that partially overlapped with the localization of RYRs with which BKCa channels are physically and functionally coupled (*SI Appendix*, Fig. S3*F*) [consistent with (23)]. Immunoblot analysis further confirmed that BKCa (KCNMA1) channel levels were unaltered by ATG5 loss (*SI Appendix*, Fig. S3*G*), consistent with our proteomic analysis. These data make it unlikely that BKCa and by extension other potassium channels (see *SI Appendix*, Fig. S3*E*) are direct substrates of ATG5-dependent neuronal autophagy and, thereby, affect AP waveform and excitability.We conclude that increased excitability and kainate-induced epileptic discharges in ATG5 cKO are caused, at least in part, by BKCa channel hyperactivity rather than alterations in BKCa channel levels.

In a final set of experiments, we wanted to dissect whether altered BKCa channel function also underlies the observed increase in presynaptic release probability in ATG5 cKO hippocampal neurons. Application of Iberiotoxin failed to restore PPF measured as a surrogate for presynaptic release probability in ATG5 cKO mice to WT controls (Fig. 4*G*). Iberiotoxin treatment also did not affect PPF of WT responses as reported before (27). Facilitated fEPSP slope/FV ratios in ATG5 cKO slices were unaffected by Iberiotoxin (*SI Appendix*, Fig. S4*B*). Analysis of fEPSP slopes/stimulation intensities revealed a partial rescue of the KO phenotype by Iberiotoxin (*SI Appendix*, Fig. S4*C*), likely due to the restoration of FV amplitudes upon BKCa channel inhibition (see Fig. 4*E*). Consistently, we found that application of Iberiotoxin caused a parallel decay of FV amplitudes (*SI Appendix*, Fig. S4*D*) and fEPSP slopes (*SI Appendix*, Fig. S4*E*) in ATG5 cKO slices, further underscoring the presynaptic nature of Iberiotoxin action.

Collectively, we demonstrate that increased excitability and kainate-induced epileptic discharges in ATG5 cKO are largely due to BKCa channel hyperactivity, while BKCa channels do not affect presynaptic release probability in our model.

## Discussion

In this study, we identify a pathway by which neuronal autophagy controls neuronal excitability and epileptogenesis, involving RYR-mediated regulation of BKCa channel function. Our findings strongly support a direct effect on neuronal excitability, although we cannot exclude the possibility that broader network interactions also contribute to the observed phenotype. We demonstrate that conditional loss of ATG5 in excitatory cortical and hippocampal neurons causes an increase in kainate-induced epileptiform bursts ex vivo and facilitates excitability of presynaptic axonal fibers in the hippocampus due to increased potassium-efflux during APs. Importantly, we find that increased excitability and kainate-induced epileptic discharges in ATG5 cKO are mediated by BKCa channel hyperactivity downstream of elevated RYR-mediated calcium entry. We also show that the peak frequency of gamma oscillations in ATG5 cKO is lower than the control. The lower gamma peak frequency point to an impaired excitation–inhibition balance between pyramidal cells and interneurons in the CA3 network (36), explained by dysfunctional BKCa-mediated hyper/repolarization in pyramidal cells (33, 34, 40). Detailed analysis of the AP waveform of ATG5 cKO pyramidal neurons revealed a shorter AP halfwidth and increased afterhyperpolarization as a consequence of increased negative AP slopes, phenotypes known to be modulated by BKCa channels (27, 30, 31, 34, 38). In agreement, we find that pharmacological blockade of RYRs or BKCa channels rescues the AP halfwidth to values that are indistinguishable between WT and cKO pyramidal neurons. We failed to detect alterations in the RMP, AP threshold, or input resistance, suggesting that conditional loss of autophagy does not have a major impact on the passive ion-leaking properties of the membrane or the function of non-AHP related ion channels. This is consistent with the observed lack of effect of cKO of ATG5 on synapse number or on pre- and postsynaptic protein levels (2). We further show using pharmacological experiments that altered BKCa channel activity in ATG5 cKO neurons does not underlie the previously observed increase in presynaptic release probability in ATG5 cKO mice caused by RYR-mediated dysregulation of axonal and presynaptic calcium homeostasis (2). Surprisingly, we also observed a small increase in membrane capacitance in ATG5 cKO neurons that was rescued by application of either Dantrolene or Iberiotoxin. The mechanism underlying the altered membrane capacitance is unclear but may reflect indirect effects of elevated intracellular calcium levels downstream of RYR function (26) on membrane surface area, dielectric properties, and/or ion distribution, among other possible changes caused by neuronal loss of ATG5.

Our findings are thus most compatible with a model whereby impaired neuronal autophagy dysregulates excitatory neuronal network activity by axonal RYR-mediated compound effects on i) presynaptic release probability ((2) and this study) and ii) axonal hyperexcitability due to calcium-triggered BKCa channel hyperactivity as demonstrated here. This model is consistent with the enrichment of BKCa channels in axons and at presynaptic sites (23), with the profound accumulation and activity of tubular ER-localized RYR within axons and at presynaptic nerve terminals under conditions of impaired autophagy (2) and with the observed nanoscale coupling of RYR and BKCa channels to rapidly regulate AP burst firing (24). Furthermore, our results agree with a previous study suggesting that BKCa channels predominantly influence neurotransmitter release under conditions of increased presynaptic excitability and high calcium influx but do not affect basal neurotransmission at CA3-CA1 synapses (27) (although this may be different at high release probability CA3–CA3 synapses; see ref. 41). That said, it is possible that other components beyond RYR and BKCa channels contribute to the phenotypic changes with respect to presynaptic release probability and hyperexcitability observed in ATG5 cKO pyramidal neurons. A limitation of our study is that we have not been able to directly record presynaptic BKCa channel function electrophysiologically. Future studies will thus be needed to fill this gap and to fully dissect the increased excitability of ATG5 cKO pyramidal neurons.

The findings reported here are compatible with and provide a possible molecular explanation for the association of impaired autophagy with increased excitability (42), neuronal network hyperactivity, and epileptogenesis (14, 17–22). In many of these studies excitatory hyperactivity and epilepsy have been linked to facilitated mTORC1 signaling, a pathway that represses autophagy. For example, it is well established that loss of function mutations in genes encoding for the mTORC1 repressing tuberous sclerosis complex (TSC) underlie focal epilepsy in animal models and in human patients. Neurodevelopmental forms of epilepsy have also been found to be caused by hyperactive mTOR mutations (43) and by loss-of-function of PI3KC2β (44), another repressor of mTORC1 signaling, among others. How hyperactive mTORC1 signaling and/or impaired autophagy impact on neuronal excitability or induce epileptogenesis in many cases has not been analyzed. We hypothesize that RYR-mediated activation of BKCa channel function under conditions of impaired autophagy contributes to neuronal hyperexcitability and network dysfunction. Our findings thereby extend the known association of loss- as well as gain-of-function mutations in the BKCa channel with various types of neurological disorders beyond epilepsy including movement disorders, as well as neurodevelopmental and cognitive diseases (29, 33, 34, 38). Given that autophagic activity declines with age (1, 45) it is tempting to speculate that pharmacological targeting of BKCa channels, possibly together with approaches to boost autophagy (46, 47), may serve as a possible therapeutic approach to combat aging-associated memory decline.

## Materials and Method

Additional Methods are described in *SI Appendix*.

### Animal Experiments.

All animal experiments were reviewed and approved by the ethics committee of the “Landesamt für Gesundheit und Soziales” (LAGeSo) Berlin and were conducted according to the committee’s guidelines. Mice were housed under 12/12-h light/dark cycle and up to five animals per cage, with access to food and water ad libitum. ATG5lox/lox [B6.129S-ATG5tm1Myok (RBRC02975)] mice were received from the RIKEN BioResourceCenter (BRC, Ibaraki, Japan) (48). To delete ATG5 in excitatory neurons in the neocortex and hippocampus, ATG5flox/flox mice were crossed with an Emx1-Cre line (49) generating ATG5flox/-3EMX1-Cre mice (first generation). Mice were monitored daily in their home cage and in reported cases, measures were taken to avoid suffering for affected individuals. To avoid suffering and loss of ATG5 cKOs animals, mice were used up to 8 wk of age. Genotypes were determined by PCR analysis from genomic DNA obtained from ear biopsy before the experiments and specimens were regenotyped DNA obtained from tail cuts spared after mice were killed. Genotyping was performed using primers P47 (GAATATGAAGGCACACCCCTGAAATG), P48 (ACAACGTCGAGCACAGCTGCGCAAGG), and P49 (GTACTGCATAATGGTTTAACTCTTGC) to detect the ATG5wt and ATG5lox alleles and TM63 (CCGGGCTGCCACGACCAA) and TM64 (GGCGCGGCAACACCATTTTT) to detect the Cre allele. For electrophysiological experiments, 6- to 8-week-old ATG5 cKO and corresponding control mice of both genders were used.

### Slice Preparation for Field Recordings.

Mice were decapitated after cervical dislocation and the brains quickly extracted into preoxygenated (95% O2/5%CO2) ice-cold dissection artificial cerebrospinal fluid (ACSF) containing: 2.5 mM KCl, 1.25 mM NaH_2_PO_4_, 24 mM NaHCO_3_, 1.5 mM MgS0_4_, 2 mM CaCl_2_, 25 mM glucose, 250 mM sucrose (pH 7.35 to 7.40). Brains were cut along the middle line and sagittal brain slices (350 µm thick) were prepared from each hemisphere in oxygenated dissection ACSF at 2 to 4 °C using Vibroslicer (Leica, VT 1200S). After preparation, slices were recovered for at least 1.5 h at room temperature (22 to 24 °C), in a resting chamber (Harvard apparatus, BSC-PC) filled with ACSF containing 120 mM NaCl instead of 250 mM sucrose. Some slices after 1 h of recovery were transferred to incubate with RYR inhibitor Dantrolene (30 µM) for 2 h, or just DMSO in ACSF (1:1,000) as control treatment. Recordings were performed in a submerged recording chamber (Warner instruments RC-27L) filled with continuously oxygenated ACSF with a solution exchange of 3 to 5 mL per min at room temperature. Slices were placed on mesh-like insert in the chamber and fixed with slice support under an upright microscope (Olympus, BX61WI) to position stimulating and recording electrodes. Glass stimulating (1 to 1.5 MΩ) and recordings (1.5 to 2.5 MΩ) electrodes filled with ACSF were prepared from glass capillaries (Hilgenberg) using micropipette puller Sutter P- 1000 (Sutter Instruments). The data were recorded at a sampling rate of 10 kHz, low-pass filtered at 3 kHz using EPC9 amplifier and analyzed using Patch Master software (Heka Elektronics).

### Recordings of fEPSPs in the CA1 Area.

For fEPSP recordings stimulating electrode was placed in *Stratum radiatum* of proximal CA1 area (close to CA2) and basal stimulation of 0.2 ms electrical stimuli were delivered at 0.05 Hz at the stimulation intensity, which induced approximately 30% of the maximal responses. The recording electrode was placed at the same depth in *S. radiatum* of the distal part of CA1, approximately 500 μm away from the stimulating electrode (*SI Appendix*, Fig. S1*E*) and slowly advanced until the responses were saturated at the given stimulation intensity. Recordings were performed in presence of GABAR antagonist Picrotoxin (50 µM) and NMDAR antagonist AP5 (50 µM), while CA3 - CA1 connections were dissected (in CA2 Area) using sharp blade to prevent spontaneous epileptiform activity. After stable baseline recordings of at least 10 min, input/output stimulus response curves were made as a measure of basal excitatory synaptic transmission. Slopes of the fEPSP were plotted against fiber volley (FV) amplitudes as a function of increasing stimulation intensity. Stimulation intensity was increased 5 µA steps until the maximal fEPSP were obtained, defined as a response with superimposed PS component on decaying fEPSP responses (*SI Appendix*, Fig. S1*E*). The amplitudes of presynaptic fiber volleys (FV) were plotted against stimulus intensity for measures of presynaptic Schaffer collateral excitability. As presynaptic Schaffer collaterals were dissected from CA3 cell bodies we assume FV amplitudes as a measure of axonal excitability, which is no longer influenced by dendritic and cellular excitability. Short-term synaptic facilitation was tested by delivering two pulses at time intervals from 10 to 500 ms at a stimulation intensity which induced approximately 20% of the maximal responses. Paired pulse facilitation (PPF) was calculated as a percentage increase of the slope of the second response as compared to the first. For short intervals (10 and 20 ms), the first fEPSPs were digitally subtracted before measurements of the second fEPSPs. Each trace measured for the stimulus response curve and paired pulse parameters is an average of 3 consecutive stimulations delivered every 20 and 30 s for stimulus response curves and paired pulse protocols, respectively.

### Recordings of PSs in the CA3 Area.

We recorded output action potential PSs from CA3 pyramidal cells as a function of the excitability of neuronal populations in CA3 area in response to antidromic stimulation. For these sets of experiments the recording electrode was placed in stratum pyramidale in CA3 area approximately 500 μm away from the stimulating electrode which was placed in *S. radiatum* of proximal CA1 area (close to CA2) in order to directly stimulate axons of CA3 cells (*SI Appendix*, Fig. S2*C*). After 10 minutes of stable baseline recordings, we recorded input output stimulus response curves with increasing stimulation intensities from 0 to 300 µA, with 30 µA steps. Each value measured is an average of three consecutive stimulations repeated every 20 s.

### Patch Clamp Whole Cell Recordings.

Hippocampal slices were prepared from 6 to 8 wk WT control and ATG5 cKO mice as previously described (2, 50). Animals were anesthetized with isoflurane and decapitated. Their brains were quickly removed and transferred to sucrose-based ice-cold artificial cerebrospinal fluid (sACSF) containing (in mM): 50 NaCl, 25 NaHCO3, 150 Sucrose, 10 Glucose, 2.5 KCl, 1 NaH2PO4, 7 MgCl2, 0.5 CaCl2. Tissue blocks containing the hippocampus were mounted on a VT1200SVibratome (Leica, Germany) and horizontal slices of 300 µm thickness were cut.

After preparation slices were recovered in an interface resting chamber in ACSF containing (in mM): 119 NaCl, 26 NaHCO3, 10 Glucose, 2.5 KCl, 1 NaH2PO4, 1.3 MgCl2, 2.5 CaCl2 (pH 7.4; 285 to 300 mOsm; ~1 ml/min) at physiological temperature (~35 °C) for at least 1 h before recordings. Recordings were performed in a submerged chamber filled with ACSF with a solution exchange of 3 to 5 mL per min, heated to 32 to 34 °C.

Whole cell patch clamp recordings were performed in CA1 pyramidal cells in order to assess the excitability of the cells as previously described (51). Cells were recorded in current clamp mode with a K-gluconate–based intracellular solution containing (in mM) K-gluconate (120), HEPES (20), KCl (3), NaCl (7), MgATP (4), NaGTP (0.3), and phosphocreatine (14), adjusted to pH 7.3 with KOH. After measuring the membrane potential, the membrane potential was manually adjusted to −60 mV (not corrected for liquid junction potential, LJP: approx. −15 mV) by continuous somatic current injection. Membrane-response and action potential (AP) firing were measured by applying depolarizing current steps (duration = 1 s, increment = 40pA). For input–output curves, the number of APs was plotted with the injected current. Input resistance was detected by measuring the voltage deflection of a hyperpolarizing current pulse. AP threshold refers to the membrane potential at which an AP is triggered (5% of the maximal AP slope). AP halfwidth represents full width of the AP measured at half maximum amplitude of the AP. Maximal number of spikes refers to the Maximal firing rate during 1 s-depolarization pulse, before cells went into depolarization-block. Afterhyperpolarization (AHP) describes the amplitude of negative deflection after the AP, measured from the AP threshold. The slope ratio was calculated by dividing the maximal positive slope with the maximal negative slope. Maximal positive and negative slopes were detected at the second AP in the current injection step after rheobase. Capacitance was calculated with a test pulse (4 mV) in voltage clamp configuration. The integrated transient charge, measured until the steady state, was divided by the voltage clamp size. For experiments with Dantrolene (30 µM), the slices were preincubated at least 2 h with the drug in the interface chamber, before the slices were transferred to the recording chamber and were recorded in the presence of Dantrolene. Iberiotoxin (200 nM) was directly applied into the recording chamber and slices were incubated at least for 15 min before recordings started.

### Recording of Gamma Oscillation and Interictal Discharges.

Corresponding control mice or ATG cKO mice of age 6 to 8 wk were decapitated following isoflurane anesthesia. Brains were transferred to ice-cold sucrose-based ACSF containing the following (in mM): 87 NaCl, 2.5 KCl, 3 MgCl_2_.6H_2_O, 0.5 CaCl_2_, 10 Glucose, 50 Sucrose, 1.25 NaH_2_PO_4_, and 26 NaHCO_3_ (pH 7.4). Horizontal slices (400 μm thick) of ventral to mid-hippocampus were cut using a vibratome (VT1200S, Leica) and stored in an interface chamber perfused with standard ACSF containing the following (in mM): 119 NaCl, 2.5 KCl, 1.3 MgCl_2_, 2.5 CaCl_2_, 10 Glucose, 1 NaH_2_PO_4_, and 26 NaHCO_3_, pH 7.4, 290 to 310 mosmol/l (32 to 34 °C, flow rate ∼1 ml/min). ACSF solutions were equilibrated with carbogen (95% O_2_, 5%CO_2_). Slices were allowed to recover for at least 1.5 h after preparation. Recordings were performed in the same interface chamber in standard ACSF at 32 to 34 °C perfused at a rate of ~1 ml/min. For LFP recordings, glass microelectrodes (tip diameter ∼5 to 10 μm; resistance: 0.2 to 0.3 MΩ) were filled with ACSF before use. Extracellular LFP (local field potential) signals were amplified 1,000×, and using a Multiclamp 700A amplifier (Molecular Devices). Signals were low-pass filtered at 4 kHz (for LFP recordings) and 4 kHz (for whole cell recordings) using the built-in Bessel filter of the amplifier and digitized at 10 kHz with 16-bit resolution analog-to-digital converter (BNC-2090 board; National Instruments). Data were sampled and stored using Igor Pro (Wavemetrics). Gamma oscillations were induced in slices using kainate model (52). The slices were perfused with 100 nM Kainate for ~30 min followed by 300 nM kainate for ~1 h 30 min. The kainate-model of induction of interictal discharges has been widely used (53). As a measure of slice quality, sharp-wave ripples were observed spontaneously in all the slices before the application of kainate. In a subset of experiments (6 slices), following interictal discharges, 100 to 200 nM Iberiotoxin was perfused onto the slices for the at least 30 min.

### Data Analysis and Statistics.

SigmaPlot (Systat Software, Inc.) was used for electrophysiological data analyses except gamma oscillations and interictal discharges (see below). Data are depicted as mean ± SEM. The number of tested slices (n) and mice (N) are indicated in the legends. Data curves were statistically evaluated using ANOVA with repeated measures and the significance of the effects was further explored using the Bonferroni *t* test post hoc analysis (significance depicted over a line encompassing the curve). Statistically significant differences between WT and ATG5 cKO groups are indicated as ***P < 0.001; **P < 0.01; *P < 0.05, while between different pharmacological conditions as ^+++^*P* < 0.001; ^++^*P* < 0.01; ^+^*P* < 0.05. Two-tailed unpaired Student´s *t* test was used for statistical comparisons between two groups.

For the analysis of gamma oscillations and interictal discharges the spectral components of the LFP were determined using a pwelch function with custom routines written in Matlab. The amplitudes of the interictal discharges were quantified using a threshold-based method. The IEI were calculated from the peaktimes of the detected interictal discharges. Statistical comparison was performed in Matlab (The Mathworks, Natick, MA) or GraphPad Prism Version 7 (GraphPad Software, Inc.). Data are reported as means ± SEM, or medians. For display boxplots are used, where the margin of error is given as the 10 and 90 percentiles (whiskers), along with the median (notch). Statistical comparisons were performed using the two-tailed unpaired or paired (Student’s) *t* test, the Mann–Whitney *U* test, the two-sample Kolmogorov–Smirnov test, or by the proportions test.

## Supplementary Material

Appendix 01 (PDF)

## Data Availability

All study data are included in the article and/or *SI Appendix*.
