# Supplementary material for: Neuronal autophagy controls excitability via ryanodine receptor–mediated regulation of calcium-activated potassium channel function
Source: Proc Natl Acad Sci U S A. 2025 Apr 23;122(17):e2413651122. doi: 10.1073/pnas.2413651122 (PMC12054804; doi:10.1073/pnas.2413651122)
Supplement: Supplementary file 1 — Appendix 01 (PDF) [file pnas.2413651122.sapp.pdf]

Supporting Information for

Neuronal autophagy controls excitability via ryanodine receptor-mediated regulation of calcium-activated potassium channel function

Gaga Kochlamazashvili, Aarti Swaminathan, Alexander Stumpf, Amit Kumar, York Posor, Dietmar Schmitz, Volker Haucke and Marijn Kuijpers

Corresponding authors: Volker Haucke, Marijn Kuijpers, and Dietmar Schmitz

This PDF file includes:

Supporting Text: Extended Methods, Supplemental Reference
Figures S1 to S4

Supporting Information Text

Extended Methods

Neuron culture preparation

Neuronal cultures were prepared by surgically removing the hippocampi from postnatal mice at p1-3 (hippocampus) or p4-7 (cerebellum), followed by trypsin digestion to dissociate individual neurons as described (1). Hippocampal cells were plated on poly-L-lysine coated coverslip and 2 mL of plating medium (basic medium (MEM; 0.5% glucose; 0.02% NaHCO3; 0.01% transferrin) containing 10% FBS, 2 mM L-glutamine, insulin and penicillin/streptomycin) was added 1 h after plating. After one day in vitro (DIV1) 1 mL of plating medium was replaced by 1 mL of growth medium (basic medium containing 5% FBS; 0.5 mM L-glutamine; 2% B27 supplement; penicillin/ streptomycin) and on DIV2 1 mL of growth medium was added. AraC was added to the culture medium to limit glial proliferation. The cerebellar granule cell (CGN) cultures used for the multiplexed SILAC are grown in poly-L-lysine coated dished containing 2 mL of Neurobasal (NB) medium supplemented with 25mM KCl . To initiate homologous recombination in neurons from floxed animals expressing a tamoxifen-inducible Cre recombinase cultured neurons were treated with 0.3 µM (Z)-4-hydroxytamoxifen (Sigma) immediately after plating.

Multiplexed SILAC and mass spectrometry analysis

The Silac data in figure S3 is extracted from previous work (1). In short, CGN WT and KO cultures (1.5-1.7x106 cells per culture) were grown in custom-made lysine and arginine-free NB (Life technologies) to which “medium” (M) variants D4-lysine/13C6-arginine (Lys4/Arg6) or “heavy” (H) variants 13C615N2-lysine/13C615N4-arginine (Lys8/Arg10) were added. Neurons were harvested and lysed after 14 days and mixed together as pairs of time-matched WT and KO sets. Forty micrograms of protein in Laemmli sample buffer from each time point was separated on 4%–15% SDS–PAGE, each lane was then cut into 15 slices, and in-gel tryptic digestion was performed. Tryptic peptides were analyzed by a reversed-phase capillary liquid chromatography system (Ultimate 3000 nanoLC system; Thermo Scientific) connected to an Orbitrap Elite mass spectrometer (Thermo Scientific). Identification and quantification of proteins were performed using MaxQuant (version 1.5.1.0) software. Data were searched against the Uniprot mouse protein database. To exclude the possibility of a specific labeling type affecting the experimental outcome, the labeling (heavy or medium type) was varied between the WT and KO samples in the four biological replicates. The graph includes the potassium channels detected in at least two biological replicates.

Immunostaining of hippocampal neurons in culture

Neurons were fixed on DIV 16 with 4% paraformaldehyde (PFA)/4% sucrose in phosphate-buffered saline (PBS) for 15 min at room temperature (RT), washed and incubated with primary antibodies in PBS containing 10% normal goat serum (NGS) and 0.3% Triton X-100 (Tx) overnight at 4 degrees. Coverslips were washed three times with PBS (10 min each) and incubated with corresponding secondary antibodies for 1 hour. Finally, coverslips were washed three times in PBS and mounted in Immumount. All acquisition settings were set equally for all groups within each immunostaining. Image processing and quantitative analysis was performed in ImageJ.

Immunoblot analysis of mouse brain extracts

Brain tissue was homogenized in lysis buffer (20 mM Hepes-KOH, pH 7.4, 100 mM KCl, 2 mM MgCl2, 1% Triton X-100, supplemented with 1 mM PMSF and mammalian protease and phosphatase inhibitor mixture) using a glass/ Teflon homogenizer. Lysates were incubated 30 min on ice before centrifugation at 17,000g for 10 min at 4 °C and protein concentrations determined by Bradford or BCA assay. Equal concentration of lysates in Laemmli sample buffer were boiled for 5 min. Between 20 and 60 µg protein was resolved by SDS–PAGE and immunoblotting was done on nitrocellulose membranes. Membranes were incubated with the primary antibodies at 4°C overnight. On the next day, bound primary antibodies were detected by incubation with IRDye 680/800CW-conjugated secondary antibodies via the Odyssey Fc Imaging system (LI-COR Biosciences).

Supplemental Reference

1. M. Kuijpers *et al.*, Neuronal Autophagy Regulates Presynaptic Neurotransmission by Controlling the Axonal Endoplasmic Reticulum. *Neuron* **109**, 299-313 e299 (2021).

Supplementary figures

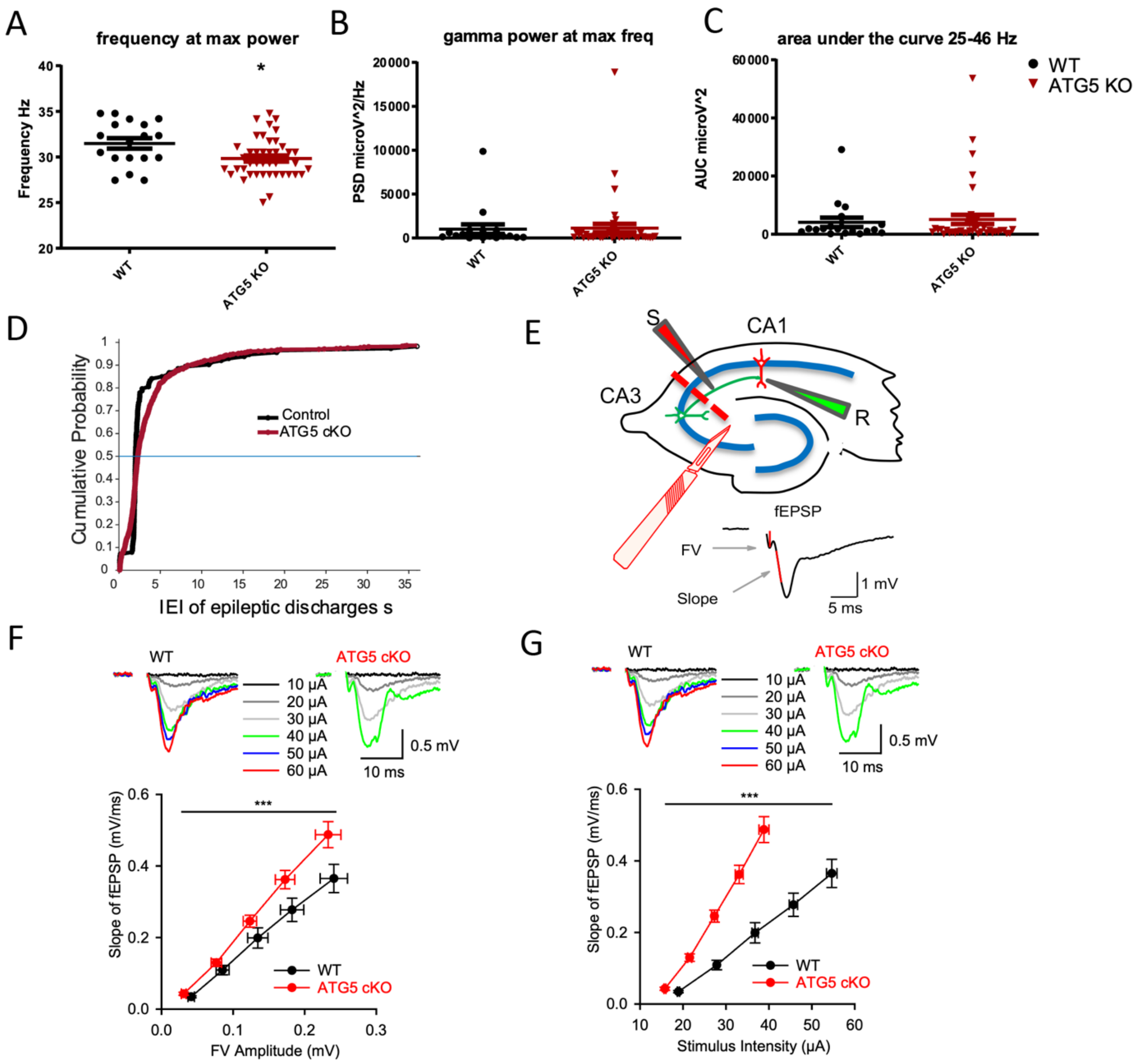

Figure S1 | Increased epileptiform bursts and excitatory neurotransmission in autophagy-deficient ATG5 cKO mice. (A-C) Attributes of gamma oscillation in wildtype control (black) and ATG5 cKO (red) mice (control n= 18, ATG5 cKO n=43; Figure 1A) **(A)** Frequency at maximum power of 30s gamma oscillation trace. Control 31.5 ±0.57 Hz vs ATG5 cKO 29.84 ±0.33 Hz; p = 0.011, unpaired t-test. **(B)** Gamma power at maximum frequency of 30s gamma oscillation trace. Control 1012 ±545.3 μV^2/Hz vs ATG5 cKO 1131 ±473.8 μV^2/Hz; p = 0.9313, Mann Whitney U-test. **(C)** Area under the curve (25-46 Hz) of PSD of 30s gamma oscillation trace. Control 4116 ±1633 μV^2 vs ATG5 cKO 5089 ±1575 μV^2; p = 0.7960, Mann Whitney U-test. **(D)** Cumulative probability distributions of inter-event intervals of epileptic discharges in slices from wildtype control (n=113 events; 2 out of 25 slices analyzed displayed epileptiform activity) and ATG5-cKO (n=727 events; 6 out of 23 slices analyzed displayed epileptiform activity) mice. (Kolmogorow-Smirnow (KS)-test, p=0.0002). See related Fig. 1A-C and legend. **(E)** Schematic illustration of the hippocampal slice with stimulating (red) and recording (green) electrodes placed in *stratum radiatum* of the CA1 region to obtain fEPSP. Representative example trace of maximal fEPSP (Insert) and measurements of presynaptic *fiber volley* (FV) amplitude and slope of fEPSPs are indicated by arrows. Recordings were performed in presence of GABAR antagonist Picrotoxin (50µM) and NMDAR antagonist AP5 (50µM). To prevent spontaneous epileptiform activity readily inducible in CA3 area when GABAR are inhibited, CA3 -CA1 connections were dissected using sharp blade. **(F)** Basal excitatory neurotransmission is facilitated in ATG5 cKO mice. Facilitated slope/FV ratios recorded

in CA1 area in ATG5 cKO mice (Two Way RM ANOVA, $p<0.001$), indicating increased basal neurotransmission in CA1 area. The number of tested slices n and mice N are reported in Figure 1D. **(G)** Facilited fEPSP-slopes in ATG5 cKO mice at similar stimulation intensities (Two Way RM ANOVA, $p<0.001$), indicating compound increase in basal excitatory neurotransmission (Figure S1F) and FV excitability (Figure 1D) of Schaffer collaterals. The number of tested slices n and mice N are reported in Fig. 1D.

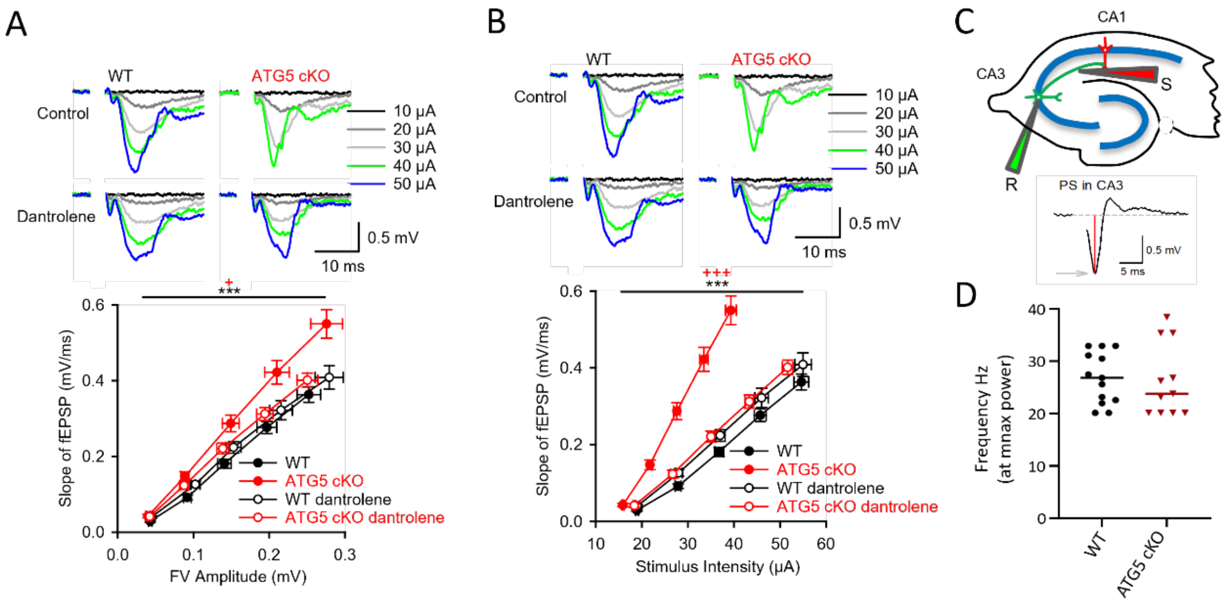

Figure S2 | Rescue of elevated basal neurotransmission by RyR inhibition. (A) Facilitated slope/FV ratios recorded in CA1 area in ATG5 cKO mice, indicating increased basal neurotransmission in the CA1 area (Two-Way RM ANOVA, p<0.001). Facilitated slope/FV ratios in ATG5 cKO mice are rescued by the RyR inhibitor Dantrolene (Two-Way RM ANOVA, p=0.026). RyR inhibition has no effect on WT responses, compared to untreated WT slices (Two Way RM ANOVA, p=0.269) and WT vs ATG5 cKO slices treated with dantrolene show no significant difference between gentypes (Two Way RM ANOVA, p=0.339). The number of tested slices n and mice N are reported in Figure 2A. **(B)** Facilitated fEPSP-slopes in ATG5 cKO mice, indicating compound increase in basal excitatory neurotransmission (A) and FV excitability (Figure 2A) (Two Way RM ANOVA, p<0.001). Facilitated fEPSP-slopes in ATG5 cKO mice are rescued by application of the RyR inhibitor dantrolene (Two Way RM ANOVA, p<0.001). RyR inhibition has no effect on WT responses, compared to untreated WT slices (Two Way RM ANOVA, p= 0.129) and WT vs ATG5 cKO slices treated with Dantrolene show no significant difference between genotypes (Two Way RM ANOVA, p= 0.588). The number of tested slices n and mice N are reported in Figure 2A. **(C)** Schematic illustration of the hippocampal slice with stimulating (red) and recording (green) electrodes placed in *stratum radiatum* and *stratum pyramidale* of the CA3 region to obtain CA3-PSs induced by antidromic stimulation. Representative example of maximal PS (Insert) and measurement of PS amplitude is indicated by arrow. **(D)** Gamma oscillation recording in wildtype control (black) and ATG KO (red) mice (control n= 13, ATG KO n=11) with Dantrolene (30µM) incubation of slices. Frequency at maximum power of 30s gamma oscillation trace. Control 26.81 ± 1.36 Hz vs ATG KO 26.36 ± 2.09 Hz; p = 0.7196, Mann Whitney U-test.

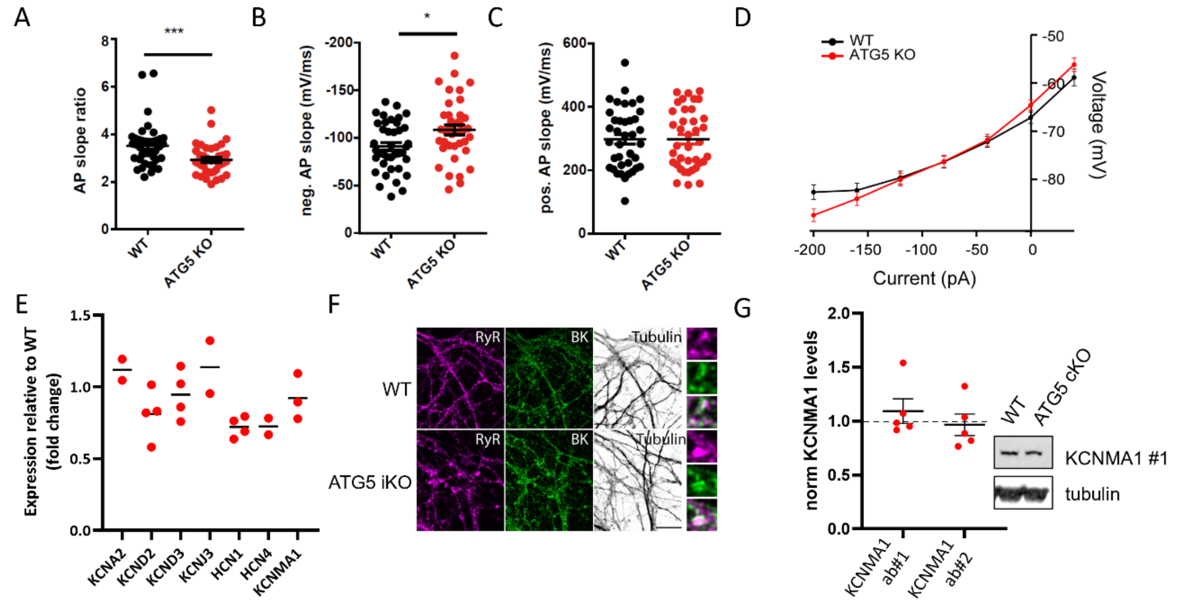

Figure S3 | Altered AP waveform in ATG5 KO hippocampal neurons is not due to altered BK channel levels or localization. (A) AP slope ratio (negative slope /positive slope) is reduced in ATG5 deficient cells; Mann-Whitney test: *p*= 0.0004. (B) Negative slope (decay phase) is higher in ATG5 KO cells, Mann-Whitney-test *p*= 0.023. **(C)** Positive slope (rising phase) of the AP is not changed in ATG5 deficient cells. Mann-Whitney-test *p*= 0.91. WT n= 39, ATG5 KO n =38. **(D)** IV curves (intensity of injected current plotted against the resulting voltage deflection in current clamp configuration) was unchanged in ATG5 deficient cells. WT n=25, ATG5 n=25; 1-way ANOVA p= 0.9758. **(E)** Comparisons of ATG5-iKO/WT SILAC ratios obtained from DIV14 CGN cultures. Graph includes potassium channels detected in at least two out of four MS/MS experiments. n, indicated in graph. Black lines represent mean. **(F)** Immunostainings in WT and ATG5-iKO hippocampal neurons show ryanodine receptor (RYR) accumulation. BK immunostaining is not changed in ATG5-iKO and partially overlaps with accumulated RYR (enlarged regions on the right). Scalebar= 10µm **(G)** Analysis and representative example of immunoblots of WT and ATG5-cKO brain lysates showing no change in KCNMA1 (BK channel) level. Data points show the protein level change of the indicated proteins normalized to the housekeeping gene tubulin. The mean values for the controls are set to 1. n=5; one-sample t -test. Error bars represent mean +/-SEM.

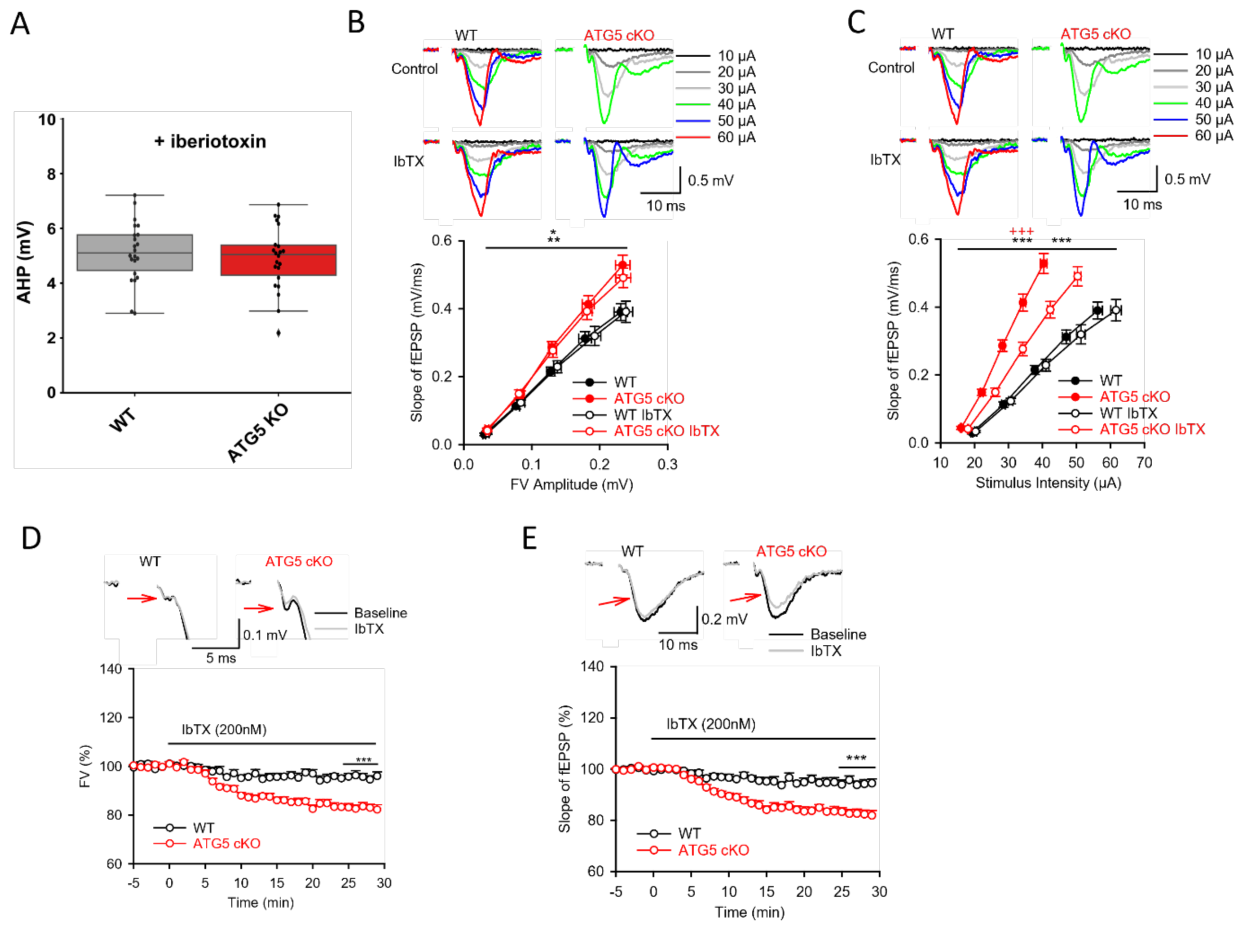

Figure S4 | Rescue of elevated basal neurotransmission by BKCa channel inhibition. (A) Peak AHP is not significantly different in WT and ATG5 KO slices treated with the BKCa channel blocker Iberiotoxin (200nM). p-value=0.53 WT n=22; ATG5 KO n=22 **(B)** Facilitated slope/FV ratios recorded in CA1 area in ATG5 cKO mice, indicating increased basal neurotransmission in the CA1 area (Two Way RM ANOVA, $p<0.001$). Facilitated slope/FV ratios in ATG5 cKO mice are not affected after application of the BKCa antagonist Iberiotoxin (Two Way RM ANOVA, p=0.099). Iberiotoxin has no effect on WT slope/FV ratios, compared to untreated WT slices (Two Way RM ANOVA, p=0.650) and WT vs ATG5 cKO slices treated with Iberiotoxin show significant difference between genotypes (Two Way RM ANOVA, p=0.013). The number of tested slices n and mice N are as reported in Figure 4E. **(C)** Facilitated fEPSP-slopes in ATG5 cKO mice, indicating compound increase in basal excitatory neurotransmission (Figure S4B) and FV excitability (see Figure 4E) (Two Way RM ANOVA, $p<0.001$). Facilitated fEPSP-slopes in ATG5 cKO mice are partially rescued after application of the BKCa antagonist Iberiotoxin (Two Way RM ANOVA, $p<0.001$). Iberiotoxin has no significant effect on WT responses, compared to untreated WT slices (Two Way RM ANOVA, p= 0.452) and WT vs ATG5 cKO slices treated with Iberiotoxin show significant difference between genotypes (Two Way RM ANOVA, p=<0.001). The number of tested slices n and mice N are reported in Figure 4E. **(D)** Iberiotoxin reduced FV amplitudes in ATG5 cKO mice recorded in the CA1 region. The profiles of monitoring FV amplitudes and administration of the BKCa antagonist Iberiotoxin (200nM) show that, Iberiotoxin has minor effect on WT responses, while ATG5 cKO responses were considerably reduced to 82,90 % (t-test between genotypes; $p<0.001$). Inserts: Representative fEPSPs before (baseline) and 25-30 min after application of the BKCa antagonist Iberiotoxin in control and ATG5 cKO mice. FV amplitudes were measured in this experiment as indicated by the arrows. The mean amplitude of FV recorded 5 min before Iberiotoxin application was taken as 100%. Data represent mean + standard error of the mean (SEM). The number of tested slices n and mice N are as reported in Figure 4E. **(E)** Iberiotoxin reduced slopes of fEPSP in ATG5 cKO mice recorded in the CA1 region. The profiles of basal synaptic transmission (Slope) and administration of the BKCa antagonist Iberiotoxin (200nM), show that Iberiotoxin has a minor effect on WT responses, while ATG5 cKO responses were considerably reduced to 82.56% (t-test between genotypes; $p<0.001$). Note that the reduction in the slopes of fEPSPs in ATG5 cKO mice is proportional to the reduced FV amplitudes, indicating the presynaptic nature of the Iberiotoxin effects. Inserts: Representative fEPSPs before (baseline) and 25-30 min after application of the BKCa antagonist

Iberiotoxin in control and ATG5 cKO mice. fEPSP slopes were measured in this experiment as indicated by the arrows. The mean slope of fEPSPs recorded 5 min before Iberiotoxin was applied is taken as 100%. Data represent mean + standard error of the mean (SEM). The number of slices n and mice N is indicated in the legend to Figure 4E.
